# How to Measure/Calculate Radiation Dose in Patients?

**DOI:** 10.1007/s00270-021-02772-x

**Published:** 2021-03-03

**Authors:** Reinhard Loose, Michael Wucherer

**Affiliations:** Institute of Medical Physics, Paracelsus Medical School, Hospital Nuremberg, Prof.-Ernst-Nathan-Str. 1, 90419 Nuremberg, Germany

## Abstract

Patients in fluoroscopically guided interventions (FGI) may be exposed to substantial radiation dose levels (SRDL). The most commonly reported adverse reactions are skin injuries with erythema or necrosis. It is therefore important for the interventional radiologist to know deterministic effects with their threshold doses. If possible all relevant modality parameters should be displayed on the interventionalists screen. Dosimetric parameters should be displayed in digital imaging and communications in medicine (DICOM) units and stored as DICOM Radiation Dose Structured Report (RDSR). The peak skin dose (PSD) is the most relevant risk parameter for skin injuries. Dose management systems (DMS) help optimising radiation exposure of patients. However, their calculation of skin dose maps is only available after a FGI. Therefore, dose maps and PSD should preferably be calculated and displayed in real time by the modality.

## Introduction

Patient exposure in fluoroscopically guided interventions (FGI) spans a wide dose range and can reach levels at which deterministic effects may occur. The reasons for this include the variety of modalities used and the wide range of interventional procedures. It is therefore important in complex interventions with substantial radiation dose levels (SRDL) to be able to measure or estimate the patient dose. This dose information should not only be available after a procedure by means of the recorded exposure parameters, but also to the interventionalist during a FGI.

The following article essentially refers to stationary angiography systems on which complex interventions e.g. in radiology, neuroradiology, cardiology or vascular surgery can be performed. Patient exposure in FGI depends on many patient, procedure and modality related parameters like kV, mAs, filtration, detector entrance dose rate, pulse rate, number of images, image processing, fluoroscopy time, geometric properties of the modality and field of view (FOV) [1, 2]. The DICOM radiation dose structured report (RDSR) is available in most new angiographic systems and enables a more detailed analysis of all exposure parameters from fluoroscopy and radiographic images or cine series [3].

For all aspects of quality assurance and dose management, the involvement of a medical physics expert (MPE) is therefore imperative in accordance with the European Directive 2013/59/EURATOM (EU-BSS) [4], especially for procedures with a higher dose such as computed tomography (CT) or FGI. Furthermore, FGI are the procedures with the highest risk of deterministic effects. If appropriate, these are to be reported to the competent authorities as "unintended exposures" in accordance with the national implementation of the EU-BSS.

## How to Analyse DICOM Dose Reports in FGI

About 20 years ago, exposure parameters from FGI were usually recorded manually in a Radiology or Hospital Information System (RIS/HIS) or paper based. Later, storage was provided together with the angiographic images in a picture archiving and communication system (PACS) as bitmap report. All of these recording methods allow only difficult analysis of the patient exposure. Today DICOM RDSR is available in most new angiography systems and provides an easy solution to collect dose parameters. This includes all exposure parameters for each fluoroscopic scene, all radiographic images or cine series with kV, mAs, geometrical parameters of C-arm, detector and more. Table 1 shows an excerpt of an angiography RDSR. In radiography and CT, the exposure data can be extracted relatively reliable from the DICOM image data even without RDSR. This is not the case with fluoroscopic procedures, as fluoroscopy scenes are usually not stored in the PACS. The DICOM image data therefore lack the dose contribution from fluoroscopy, which can easily exceed 50% of the total dose depending on the type of intervention. Recording and processing of patient exposure was driven by the EU-BSS which requires member states of the European Union to ensure justification and optimisation of radiological procedures and store information on patient exposure for analysis and quality assurance [4, 5]. Various commercial dose management systems (DMSs) with varying characteristics are available today [6]. In contrast to radiography and CT, complex RDSR reports are not always correctly and completely saved as DICOM objects in FGI and are not always correctly and completely evaluated by DMS providers. This is particularly important because all contributions from fluoroscopy and radiography / cine series are required to determine the total exposure of a patient. Furthermore, a complete recording of all individual radiation events is required to calculate the dose distribution on the patient's surface and to identify locations where overlapping radiation fields can lead to a high peak skin dose (PSD) and thus to potential deterministic skin injuries.

**Table 1:**
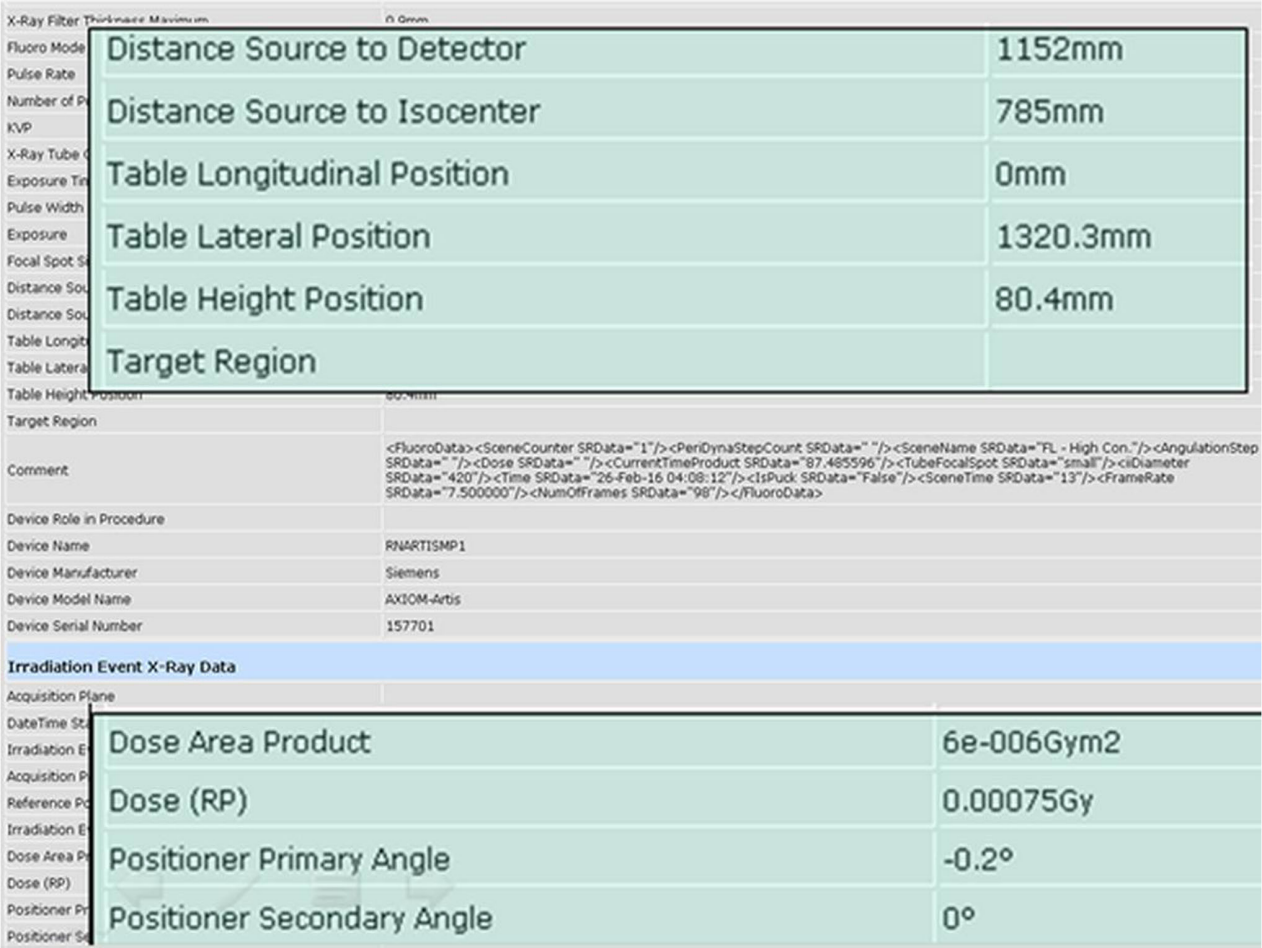
Excerpt from a DICOM RDSR with relevant modality and exposure parameters. Depending on the type of procedure a RDSR can consists of many kilobytes of exposure events

The most commonly used exposure parameters are Kerma-area product (KAP) and Air kerma at the patient entrance reference point (K_a,r_). KAP is used for diagnostic reference levels (DRLs) in most countries and is also displayed or transmitted by the manufacturers of all angiography systems. KAP is a public tag in both the Radio Fluoroscopy (RF) and X-ray angiography (XA) DICOM Service Class objects. The second most important parameter is K_a,r_, which correlates more than KAP with the skin dose, followed by the total fluoroscopy time in the number of cine series or images. KAP and K_a,r_ are usually transmitted cumulatively in RDSR for an entire examination, while the dose distribution on the body surface with PSD has to be calculated from all individual exposure events. Since dosimetric data are usually transferred to a PACS after an examination has been completed, the dose distribution is only available in a DMS after the procedure. An online display of the skin dose distribution on the modality screen during the intervention would be desirable in order to avoid high PSD by changing the projection direction and hence the skin entrance field from time to time.

## Parameters of Patient Exposure

Patient exposure to radiation can be measured directly or indirectly [7]. Direct dosimetry requires the use of dosimeters. Real time measurements are performed with ionisation chambers, diodes, metal oxide semiconductor field-effect transistors (MOSFET) and other devices. Non-real time measurements use thermoluminescent dosimeters (TLD) or optically stimulated luminescence dosimeters (OSL), other devices or film dosimetry in earlier times. Currently, most of the modern interventional systems use an ionization transmission chamber to measure the KAP and estimate K_a,r_. Some manufacturers calculate these dosimetric quantities from the modality parameters (e.g. tube output).

Direct dosimetry is time-consuming and therefore mostly limited to measurements on phantoms or patients in clinical studies. In clinical routine, indirect dosimetry with dose parameters derived from the modalities are used because of their easy availability [1].

Electronic real time dosimeters are also used for occupational dosimetry of staff members. It must be noted that these dosimeters are suitable for measuring scattered radiation, but not radiation in the primary beam, since in this case they will display incorrect dose data.

### Modality Related Exposure Parameters

**Fig. 1:**
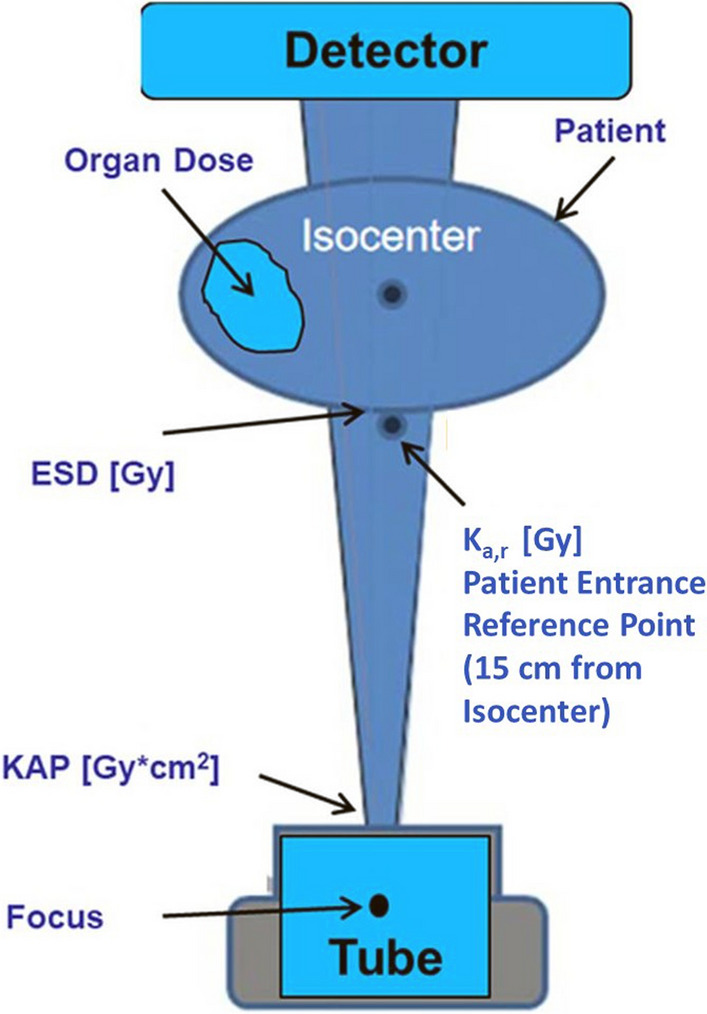
Monoplane angiography system with relevant dosimetric parameters. KAP (or DAP) = Dose Area Product, K_a,r_ = Dose at patient entrance reference point as defined by IEC [9], ESD = Entrance Surface Dose

Figure 1 shows a simple illustration of a monoplane angiography system with the relevant dosimetric parameters.

#### Kerma-Area-Product (KAP)

KAP is the integral of air kerma across the entire x-ray beam emitted from the x- ray tube (also called Dose Area Product DAP) [8]. KAP is a surrogate measurement for the entire amount of energy delivered to the patient by the beam. KAP is measured in [Gy*cm^2^] and does not include scatter.

KAP is the most important and most frequently used exposure parameter in radiographic and fluoroscopic examinations. It is available on most modalities and display and recording is required by law in many countries. Most of the DRLs in radiography and fluoroscopy are based on KAP. However, KAP does not allow an estimate about the risk of deterministic injuries to the skin or organs, as KAP is a product of dose and area and therefore large areas with a low dose or small areas with a high dose can provide identical values of KAP. For measurement of KAP an ionization chamber is placed beyond the X-ray collimators and must intercept the entire radiation field for accurate dosimetric results. Instead of measurement with ionization chambers, some manufacturers calculate KAP from modality parameters, e.g. tube output. With this procedure, it must be taken into account that a missing or incorrect calibration leads to incorrect KAP values.

#### Dose at the Patient Entrance Reference Point (K_*a,r*_)

K_a,r_ is a dose accumulated at a specific point in space relative to the fluoroscopic gantry during a procedure. The International Electrotechnical Commission (IEC) defines a patient entrance reference point as 15 cm from the isocentre of a C-arm x-ray unit on the central beam towards the focus (Fig. 1) [9]. K_a,r_ is measured in Gy. K_a,r_ is sometimes referred to as reference dose, cumulative dose, or cumulative air kerma. K_a,r_ is only a rough estimation of skin dose and not the equivalent to the skin dose. The patient entrance reference point may correspond to the skin level, a point within the patient, or a point outside of the patient. The patient entrance reference point is thus more a technical characteristic of the equipment than operational information of the exposure of a real patient. In addition, K_a,r_ does not include beam repositioning, backscatter, or the attenuation of the table. Since mid-2006, the U.S. Food and Drug Administration requires that all fluoroscopes sold in the U.S. be capable of displaying the total air kerma at the IRP.

#### Entrance Surface Dose (ESD)

The entrance surface dose or entrance skin dose (ESD) is the measure of the radiation dose [mGy] that is absorbed by the skin of a patient. Entrance skin dose includes backscatter, should include (or estimate) the attenuation of the table [10] and is either directly measurable using dosimeters or can be calculated using the modality related dose parameters [11, 12].

However ESD is a poor indicator of radiation risk as it does not account for tissue sensitivity, penetration and exposed field size. Since ESD does not include the exposed field size, ESD is a better surrogate parameter for estimating the risk of deterministic skin reactions, provided the position of the radiation field on the skin does not change.

#### Peak Skin Dose

**Fig. 2:**
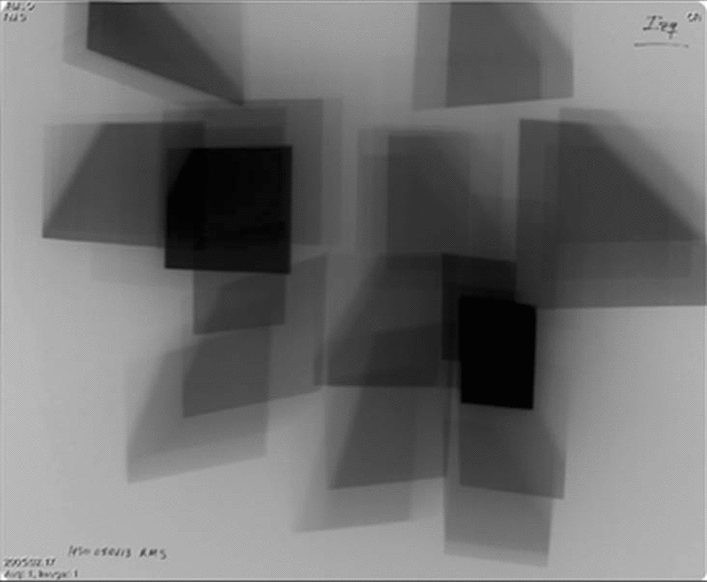
Example of skin dose distribution in cardiology procedures (measured with slow radiochromic film). These conventional films have been used for verification and dosimetry in radiotherapy. Skin dose during a conventional percutaneous coronary intervention was 0.4 Gy [2]

**Fig. 3:**
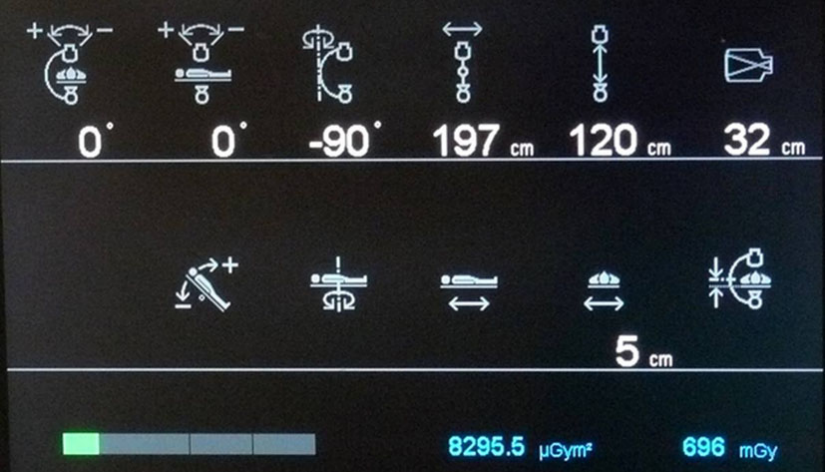
Display of geometrical and dose parameters in the interventional room with cumulative KAP and K_a,r_

**Fig. 4:**
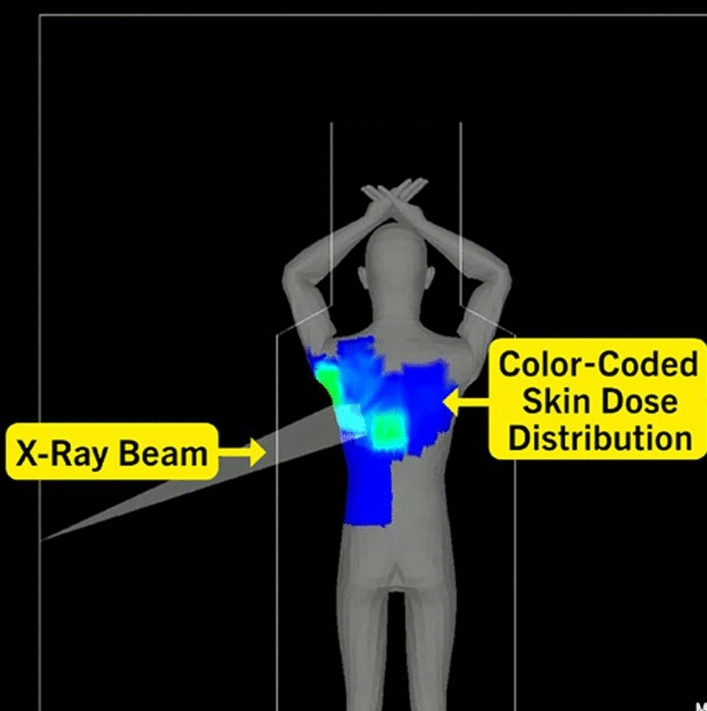
Example of a peak skin dose map from cardiology (Dose Tracking Software DTS, Canon Medical Systems). This map together with numerical dose information was generated by a DMS after the procedure [14]

PSD is defined as the highest dose at any portion of a patient’s skin during a procedure. PSD includes contributions from both the primary X-ray beam and from scattered radiation and is measured in Gy. The level and distribution of the skin dose in FGI can either be measured or calculated. In the past, measurements were often carried out with radiochromic films, the density of which is a measure of the dose and the distribution of the radiation entry fields. Figure 2 shows the dose distribution with radiochromic film of a cardiological intervention [2]. Since the introduction of the DICOM RDSR in angiography, PSD can also be calculated with certain errors. In addition to KAP and / or K_a,r_ all geometric parameters of the respective position of the C-arm, table and collimation are required in the RDSR for all radiation events (fluoroscopy and image / cine series) [13]. Figure 3 shows the relevant geometrical parameters, KAP and K_a,r_ at the modality screen. All changes in the position of the table or C-arm are recorded in individual RDSR objects together with the respective collimation and the current values of KAP and K_a,r_. From these data, a dose distribution can be calculated, represented graphically and the PSD determined. Figure 4 shows a coloured dose map during a cardiological procedure [14]. Since all geometrical data are based on parameters of the modality, large errors or unusable results arise when the patient's position relative to the table changes. In the meantime, DMS from several manufacturers can calculate dose maps and PSD and display them graphically. This always happens after the end of an intervention. It would therefore be an advantage if dose maps and PSD were calculated online by the device manufacturer and displayed live on a modality screen in the intervention room.

#### Organ Dose

The organ dose of tissue located in the radiation field can be estimated via the ESD. This requires depth-dose curves in tissue for the respective radiation quality. The attenuation in a patient by means of half-value thicknesses of the tissue can also be used as a rough estimate. As a first approximation, the half-value thickness of 3 cm in tissue (water) can be assumed for typical radiation spectra in angiography systems. The organ dose also depends on the extent to which the organ has been fully or partial exposed and, accordingly, on how the dose is distributed in the organ. Organ doses can also be determined using Monte Carlo software or conversion factors from KAP. The latter can also be used to estimate the effective dose (ED) [15].

The dose estimation is subject to many uncertainties (metrological, patient-specific). The uncertainty for the KAP is in a range of 25% that of the ESD calculated from KAP in the range of 50%. This means that uncertainties of over 50% must be expected for individual organ dose estimates. Backscatter factors depend strongly on the field size and the filtration and tube voltage and are in case of FGI around 1.4 (1.3–1.5) for water [10].

#### Effective Dose

Different tissues have varying degrees of radiosensitivity. For example, breast, bone marrow and colon are much more radiosensitive than bone surface, brain and skin. To account for this, tissue weighing factors have been developed [16, 17]. Mathematically, ED is the sum over irradiated tissues of the product of the equivalent dose and the tissue weighting factor for those tissues [18]. The unit of ED is the Sievert (Sv). It is important to note that tissue weighting factors are based on population age- and sex-specific averages, which contribute significantly to differences in individual risk. The chain below shows the relationship between absorbed dose, equivalent dose and effective dose [18].
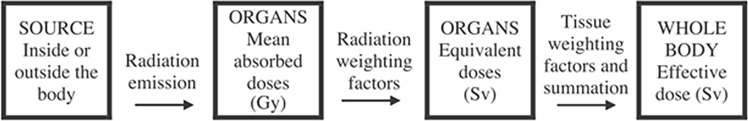


It should be noted that ED has been introduced to assess the risk of occupational exposure. The ICRP does not recommend using ED for risk calculations of individual patients. Due to the uncertainty of all parameters involved in the chain of an ED calculation, the error of the ED estimate for individual patients is significantly above 50%.

## Summary

The risk of skin radiation injuries, rarely of organ injuries, has long been known in interventional radiology. However, calculating or measuring patient doses and their distribution in organs or on the surface of the body is not an easy task. In the past, thresholds were suggested for easily available parameters such as KAP, K_a,r_ or fluoroscopy time above which deterministic reactions can be expected [7]. Once the threshold dose is exceeded, the injury becomes progressively more severe with increasing dose, although the true severity of major injuries will only become apparent weeks to months after the procedure. These thresholds are also called SRDL. Stecker et al. suggested parameters for a first SRDL notification [8]: PSD 2 Gy, K_a,r_ 3 Gy, KAP 300 Gy*cm2, fluoroscopy time 30 min.

The National Council on Radiation Protection and Measurements (NCRP) suggests a SRDL be defined at a PSD of 3 Gy or a K_a,r_ of 5 Gy [19].

In summary:

- Interventionalist should know SRDL levels with high risk of skin injuries [20]
- KAP, K_a,r_, fluoroscopy time and if possible dose maps should be displayed online on the interventionalists screen
- Dose maps and PSD should preferably be displayed in real time and not only in DMS
- The radiation entrance field should be changed from time to time in complex interventions to avoid overlapping fields with high PSD
- DMS is a helpful tool to analyse and optimise radiation protection of patients
- All measured or calculated dose parameters should be stored for analysis

## Abbreviations

CT: Computed tomography
DICOM: Digital Imaging and Communications in Medicine
DMS: Dose management system
DRL: Diagnostic reference level
ED: Effective dose
ESD: Entrance surface dose
EU-BSS: European Basic Safety Standards
FGI: Fluoroscopically Guided Intervention
FOV: Field of View
HIS: Hospital Information System
ICRP: International Commission on Radiological Protection
IEC: International Electrotechnical Commission,
KAP: Kerma-area product
K_a,r_: Air kerma at the patient entrance reference point
MPE: Medical physics expert
NCRP: National Council on Radiation Protection and Measurements
OSL: Optically stimulated luminescence dosimeter
PACS: Picture archiving and communication system
PSD: Peak Skin Dose
RDSR: Radiation Dose Structured Report
RF: Radio Fluoroscopy
RIS: Radiology Information System
SRDL: Substantial Radiation Dose Level
TLD: Thermoluminescent Dosimeter
XA: X-ray angiography
